# 303. Viral and bacterial infections among adults hospitalized with COVID-19, Coronavirus Disease 2019-Associated Hospitalization Surveillance Network, 14 states, March 2020–February 2022

**DOI:** 10.1093/ofid/ofac492.381

**Published:** 2022-12-15

**Authors:** Shah Melisa, Christopher Taylor, Kadam Patel, Jennifer Milucky, Michael Whitaker, Huong Pham, Onika Anglin, Arthur Reingold, Isaac Armistead, Kimberly Yousey-Hindes, Evan J Anderson, Andy Weigel, Libby Reeg, Erica Mumm, Susan L Ropp, Alison G Muse, Sophrena Bushey, Eli Shiltz, Melissa Sutton, Keipp Talbot, Andrea Price, Fiona P Havers

**Affiliations:** Centers for Disease Control and Prevention, Atlanta, Georgia; CDC, Atlanta, Georgia; Centers for Disease Control and Prevention, Atlanta, Georgia; Centers for Disease Control and Prevention, Atlanta, Georgia; CDC, Atlanta, Georgia; Centers for Disease Control and Prevention, Atlanta, Georgia; CDC, GDIT, Atlanta, Georgia; University of California, Berkeley, Oakland, California; Colorado Department of Public Health and Environment, Denver, Colorado; Yale School of Public Health, New Haven, Connecticut; Emory University School of Medicine, Atlanta, Georgia; Iowa Department of Public Health, Des Moines, Iowa; Michigan Department of Health and Human Services, Lansing, Michigan; Minnesota Department of Health, Saint Paul, Minnesota; New Mexico Department of Health, Santa Fe, New Mexico; New York State Department of Health, Albany, New York; University of Rochester School of Medicine and Dentistry, Rochester, New York; Ohio Department of Health, Columbus, Ohio; Oregon Health Authority, Portland, Oregon; Vanderbilt University Medical Center, Nashville, Tennessee; Salt Lake County Health Department, SLC, Utah; CDC, Atlanta, Georgia

## Abstract

**Background:**

Coinfections, both bacterial and viral, occur with severe acute respiratory syndrome coronavirus 2 (SARS-CoV-2) infection, but prevalence, risk factors, and associated clinical outcomes are not fully understood.

**Methods:**

We used the Coronavirus Disease 2019-Associated Hospitalization Surveillance Network (COVID-NET), a population-based surveillance platform to investigate the occurrence of viral and bacterial coinfections among hospitalized adults with laboratory-confirmed SARS-CoV-2 infection during March 2020 and February 2022. Patients receiving additional standard of care (SOC) molecular testing for viral pathogens (14 days prior to admission or 7 days after), including respiratory syncytial virus, rhinovirus/enterovirus (RV/EV), influenza, adenovirus, human metapneumovirus, parainfluenza viruses, and endemic coronaviruses, were included. SOC testing for clinically relevant bacterial pathogens (7 days before admission or 7 days after) from sputum, deep respiratory, and sterile sites were included. The demographic and clinical features of those with and without bacterial infections were compared.

**Results:**

Among 2,654 adults hospitalized with COVID-19 and tested for all 7 virus groups, another virus was identified in 3.1% of patients. RV/EV (1.2%) and influenza (0.4%) were the most commonly detected viruses. Half (17,842/35,528, 50.2%) of hospitalized adults with COVID-19 had bacterial cultures taken within 7 days of admission, and 1,092 (6.1%) of these had a clinically relevant bacterial pathogen. A higher percentage of those with a positive culture died compared to those with negative cultures (32.3% vs 13.3%, p< 0.001). *Staphylococcus aureus* was the most common isolate overall; *Pseudomonas aeruginosa* was the second most common respiratory isolate (Figure 1).

**Figure 1:**
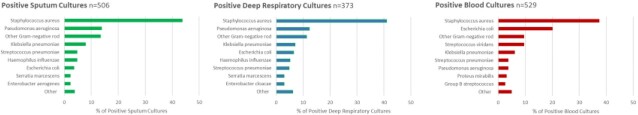
Microbial cultures from hospitalized sampled adults with COVID-19 from Coronavirus Disease 2019-Associated Hospitalization Surveillance Network (COVID-NET) from March 2020 to February 2022 with bacterial pathogens detected in sputum, deep respiratory, or blood cultures within 7 days of admission.

This figure includes 1,408 bacterial cultures from 1,066 individuals. Deep respiratory sites include endotracheal aspirate, bronchoalveolar lavage fluid, bronchial washings, pleural fluid, and lung tissue. Commensal organisms were excluded.

**Conclusion:**

Consistent with previous studies, a relatively low proportion of adults hospitalized with COVID-19 had concomitantly identified viral or bacterial infections. Identification of a bacterial infection within 7 days of admission is associated with increased mortality among adults hospitalized with COVID-19. Conclusions about the clinical relevance of bacterial infections is limited by the retrospective nature of this study.

**Disclosures:**

**Evan J. Anderson, MD**, GSK: Advisor/Consultant|GSK: Grant/Research Support|Janssen: Advisor/Consultant|Janssen: Grant/Research Support|Kentucky Bioprocessing, Inc: Data Safety Monitoring Board|MedImmune: Grant/Research Support|Medscape: Advisor/Consultant|Merck: Grant/Research Support|Micron: Grant/Research Support|NIH: Funding from NIH to conduct clinical trials of Moderna and Janssen COVID-19 vaccines|PaxVax: Grant/Research Support|Pfizer: Advisor/Consultant|Pfizer: Grant/Research Support|Regeneron: Grant/Research Support|Sanofi Pasteur: Advisor/Consultant|Sanofi Pasteur: Grant/Research Support|Sanofi Pasteur: Data Adjudication and Data Safety Monitoring Boards|WCG and ACI Clinical: Data Adjudication Board.

